# Change in Nutrient and Dietary Intake in European Children with Cystic Fibrosis after a 6-Month Intervention with a Self-Management mHealth Tool

**DOI:** 10.3390/nu13061801

**Published:** 2021-05-26

**Authors:** Joaquim Calvo-Lerma, Mieke Boon, Jessie Hulst, Carla Colombo, Inês Asseiceira, María Garriga, Etna Masip, Ine Claes, Anna Bulfamante, Hettie M. Janssens, Maria Roca, Saioa Vicente, Victoria Fornés, Laura Zazzeron, Bo van Schijndel, Sandra Woodcock, Luisa Pereira, Kris de Boeck, Carmen Ribes-Koninckx

**Affiliations:** 1 Instituto de Investigación Sanitaria La Fe—Hospital Universitari i Politècnic La Fe, 46026 Valencia, Spain; masip_etn@gva.es (E.M.); maria_roca@iislafe.es (M.R.); ribes_car@gva.es (C.R.-K.); 2 Center for Cystic Fibrosis, Department of Pediatrics, University Hospital Leuven, 3000 Leuven, Belgium; mieke.boon@uzlueven.be (M.B.); ine.claes@uzleuven.be (I.C.); kdeboeck@uzleuven.be (K.d.B.); 3 Division of Gastroenterology, Hepatology and Nutrition, Hospital for Sick Children, Toronto, ON M5G 1X8, Canada; jessie.hulst@sickkids.ca; 4 Cystic Fibrosis Center, University of Milan, IRCCS Ca ‘Granda, Maggiore Policlinico Hospital, 20122 Milan, Italy; carla.colombo@unimi.it (C.C.); anna.bulfamante@virgilio.it (A.B.); laura.zazzeron@policlinico.mi.it (L.Z.); 5 Centro de Fibrose Quística, Hospital de Santa Maria, 1649-028 Lisbon, Portugal; inesasseiceira@gmail.com (I.A.); mluisafpereira@gmail.com (L.P.); 6 Unidad de Fibrosis Quística, Hospital Universitario Ramón y Cajal, 28010 Madrid, Spain; maria.garriga@salud.madrid.org (M.G.); saioa.vicente@salud.madrid.org (S.V.); 7 Department of Pediatrics, Division of Gastro-Enterology, Erasmus MC-Sophia Children’s Hospital, University Hospital Rotterdam, 14010 Rotterdam, The Netherlands; h.janssens@erasmusmc.nl (H.M.J.); b.vanschijndel@erasmusmc.nl (B.v.S.); s.woodcock@erasmusmc.nl (S.W.); 8 Department of Pediatrics, Division of Respiratory Medicine and Allergology, Erasmus MC-Sophia Children’s Hospital, University Hospital Rotterdam, 14010 Rotterdam, The Netherlands; 9 Tauceramica Analytics, 46980 Paterna, Spain; vfornes@tauanalytics.es

**Keywords:** cystic fibrosis, nutrition, self-management, m-health, nutrients, diet, food groups, dietary habits

## Abstract

Cystic Fibrosis (CF) is a life-long genetic disease, causing increased energy needs and a healthy diet with a specific nutrient distribution. Nutritional status is an indicator of disease prognosis and survival. This study aimed at assessing the effectiveness of a self-management mobile app in supporting patients with CF to achieve the dietary goals set by the CF nutrition guidelines. A clinical trial was conducted in pancreatic insufficient children with CF, followed in six European CF centres, where the self-management app developed within the MyCyFAPP project was used for six months. To assess secondary outcomes, three-day food records were compiled in the app at baseline and after 3 and 6 months of use. Eighty-four subjects (mean 7.8 years old) were enrolled. Compared to baseline, macronutrient distribution better approximated the guidelines, with protein and lipid increasing by 1.0 and 2.1% of the total energy intake, respectively, by the end of the study. Consequently, carbohydrate intake of the total energy intake decreased significantly (−2.9%), along with simple carbohydrate intake (−2.4%). Regarding food groups, a decrease in ultra-processed foods was documented, with a concomitant increase in meat and dairy. The use of a self-management mobile app to self-monitor dietary intake could become a useful tool to achieve adherence to guideline recommendations, if validated during a longer period of time or against a control group.

## 1. Introduction

Cystic Fibrosis (CF) is a recessive genetic disease caused by a defect in the transmembrane protein that transports chloride across the cellular membrane (CFTR). Reduced chloride transport leads to thick mucosal secretions that cause duct obstruction, leading to failure in several organ systems, especially lungs and pancreas [1,2]. Chronic pulmonary infection and inflammation along with exocrine pancreatic insufficiency (present in up to 90% of the patients) account for increased energy needs, from 110 to 200% of the daily recommended intake for healthy children of the same age. According to the CF-specific guidelines, this high energy intake should be achieved by a macronutrient distribution of 20% protein, 35–40% fat and 40–45% carbohydrates [3]. Achieving and maintaining an adequate nutritional status is crucial, as this factor has shown a strong association with better lung function, which is translated into better disease prognosis and survival [2,4,5].

Up to 2016, the clinical guidelines for nutrition in CF advised the consumption of energy-dense foods and high fat intake, in order to reach the energy requirements [6]. Therefore, a common pattern in dietary habits for the CF population is still the presence of snacks or ultra-processed foods with high saturated fat or sugar contents [7,8,9]. The most recent guidelines, however, highlight that high energy intake should not be achieved at all costs but by careful selection of foods with healthy fat and low sugar content [3]. To achieve that goal, dietary counselling by CF dietitians is highly relevant, as acknowledged in the current guidelines with “high evidence” grade. The progressive increase in life expectancy in CF is the main reason to avoid life-long unhealthy diets associated with adverse outcomes in adulthood, such as obesity and possibly cardiovascular complications [8,10]. In this sense, “the nutrition paradigm has to be extended from nutrition for growth and survival to nutrition for health and well-being” [8]. The other pillar in the nutritional management of CF is pancreatic enzyme replacement therapy, consisting of oral intake of pancreatin supplements with every meal to allow nutrient digestion and absorption [11].

Despite the importance of adequate nutrition in CF, research addressing dietary patterns and nutrient intake in the CF population is limited [8], and high-impact interventional studies to improve dietary habits and nutrition quality are needed. Recently, in a European-Union-funded project (MyCyFAPP [12]), a mobile app for the self-management of nutrition and pancreatic enzyme replacement therapy (PERT) was developed in co-creation with healthcare professionals, children with CF and their families [13]. The app included different tools to support the achievement of the nutritional goals established by the recent guidelines, given the identified challenges [9], along with adjustment of the optimal dose of PERT, according to an evidence-based mathematical model [14]. The complete MyCyFAPP self-management system was evaluated in a multicentre 6-month clinical trial. After the study period, gastrointestinal-related quality of life improved significantly [15]. Moreover, the use of enzymes changed in variability in the dose, as supported by reduced IQR of the median intake up to a range of 1000–4000 LU/g fat. This finding was reported along with improved fat absorption in patients showing the poorest results at study baseline, while those with high levels of fat absorption maintained these high values after the intervention [16]. However, a remaining research question relates to the impact of the app use on dietary patterns as referred to in current guidelines.

The present study therefore aimed at assessing the change in energy and nutrient intake in European children with CF after an intervention with the newly developed mobile app (MyCyFAPP) that offered educational resources about nutrition and dietary advice and follow-up during 6 months.

## 2. Materials and Methods

### 2.1. Patients and Study Design

The 6-month, prospective, open-label, multicentre, international clinical trial protocol reported in detail the effect on the primary outcome [15]. During the study period, three study visits were scheduled, including the run-in visit (month 0) and visits after three and six months from start (visits 1, 2 and 3, respectively). At every visit, clinical and anthropometric data were collected, and patients had to complete at least three daily food records (preferably 2 weekdays and 1 weekend day) with the self-management app in the week before the visits to the hospital. The present study used the dietary and anthropometric data as secondary outcomes of a previous study design focused on assessing change in quality of life, for which a power calculation was performed [15]. Therefore, the present study can be considered as a pilot study taking into account the aim of assessing dietary and nutrient intake.

Clinically stable patients (2–18 years old) from Lisbon, Madrid, Valencia, Milan, Leuven and Rotterdam with a confirmed diagnosis of CF (sweat chloride ≥60 mEq/L and/or the presence of 2 disease-causing mutations in the CFTR gene) were invited to participate. In addition, patients had to be pancreatic insufficient (faecal elastase <200 µg/g stool) and treated with PERT. Exclusion criteria included acute infections or acute abdominal pain and recent (<3 months) onset of CFTR modulator therapy. In addition, for this part of the study, only patients who regularly used the app and appropriately entered complete food records for at least 3 days before each one of the 3 visits were considered. A food record was considered complete if at least 1000 kcal/day and 5 meals were registered per day.

The study protocol was approved by the Ethical Committees of all the participating centres. The clinical trial was registered with the Spanish Agency of Drugs and Medical Devices (Ministry of Health), reference number 645/17/EC. The trial was carried out in accordance with The Code of Ethics of the World Medical Association (Declaration of Helsinki).

### 2.2. Food Recording with the App

The interventional tool, i.e., the self-management app, was installed on a smartphone device (Motorola moto g4, XT1622) and handed to the participating families during the run-in visit. The app had a home menu with the following items: food diary, nutrition follow-up (goals), symptoms diary (health diary), nutrition educational material (living with CF) and messages among other functions (Figure S1), altogether supporting nutritional education through self-management. Periodic food recording created a nutrient intake registry that informed patients about their progress in achieving the goals, and specific nutritional education resources could be consulted at any time.

Through the “food diary” application, patients older than 12 years, otherwise their parents, were able to record food intake in a patient-friendly but detailed manner (Figure S2). The quantity of food could be indicated either by household measures of the portion size (estimated) or in grams (weighed). Patients were informed (with display bars) about the energy content of each meal and its contribution to the macronutrient categories, in percentage of the daily goal pre-set by the dietitian. Patients could re-adjust food choices or amounts to meet the goals, or at least, be aware of their degree of compliance. As per study protocol, patients had to at least complete the food record during three consecutive days in between study visits.

All the collected food records were stored by the system and displayed in the “Goals” section of the main menu, by means of interactive graphs (Figure S3). The graphs showed the evolution of energy and nutrient intake over time and compared the reported intake to daily goals. Patients were advised to check this section regularly. In parallel, data were synchronised with the “Professional web tool”, managed by the dietitians and paediatricians in the hospital, who set the goals for each patient. They also checked monthly the progress graphs of each patient (Figure S4), sent feedback via “messages” that patients received in the app and discussed the progress together during the next visit.

The app worked on the background of a food composition database, which was built with all the food items frequently consumed by European children with CF [9]. For each food item, the following information was automatically calculated: energy (kcal), protein (g), total carbohydrates (g), simple carbohydrates (sugar) (g), complex carbohydrates (g), lipids (g), monounsaturated fatty acids (MUFA) (g), polyunsaturated fatty acids (PUFA) (g), saturated fatty acids (SFA) (g), fibre (g), sodium (g), iron (mg) and calcium (mg). In addition, each food item was assigned to a food group [9]: milk and dairy, meat, fish, eggs, legumes, cereals, fruit, vegetables, nuts, oils, solid fats, ultra-processed foods, and others. All data were visible for the patients and health-care professionals in the form of graphs. To facilitate food recording, each food item in the database also included the amount in grams for the corresponding household measuring units. Then, with the app, the amount of each food could be registered in grams (if weighed) or estimated in terms of household measures by means of a display bar expressing the unit (e.g., 1 glass, 1 piece, 1 unit, 1 plate) or their possible fractions (a half, 2, 3…), which were associated with the amount in grams.

All data collected by the patients with the app were processed in real time by the calculation algorithms and stored on the project server. At the end of the study, the data repository on the server was exported into a database structured per centre, patient, day, type of meal, and food items making up the meal and its corresponding food group and nutritional facts.

### 2.3. Statistical Analysis

The exported database was merged with the electronic case report form of each patient in which clinical data were recorded (age, gender and biometry). All data are summarised as mean and standard deviation or median and 1st and 3rd quartile as appropriate. Minimum energy intake recommendation (110% of healthy age matched subjects [3]) was calculated for all the patients. Descriptive results of energy, macronutrient intake and food groups’ contribution to energy intake are presented in graphs, considering the whole cohort and per centre. Linear mixed regression models were used to assess possible associations between energy intake and nutritional status, considering age and gender. Changes in macronutrient distribution from v1 (month 0) to v3 (month 6) were assessed by means of a linear mixed regression model.

## 3. Results

Of the 170 participants in the clinical trial (clinical and demographic characteristics described by Boon et al. (2020) [15]), only those with appropriately entered food records were considered, i.e., a subset of 84 subjects with a median age of 7.8 years (5.0, 10.5) and equal sex distribution (49.8% male).

Nutritional status indicators were expressed as median (1st, 3rd quartile) z-scores. At study baseline (v1), z-scores of −0.23 (−0.85, 0.32) for weight-for-age, −0.31 (−0.81, 0.47) for height-for-age and −0.29 (0.35, −0.91) for BMI-for-age were obtained. At the end of the study, these z-scores had not statistically significantly changed: −0.24 (−0.8, 0.34) for weight-for-age, *p* = 0.68; −0.11 (−0.66, 0.62) for height-for-age, *p* = 0.26 and −0.32 (0.31, −0.94) for BMI-for-age, *p* = 0.82). Neither the change in weight-for-age, height-for-age nor BMI-for-age showed significant associations with the age of the subjects.

### 3.1. Change in Energy Intake

**Figure 1:**
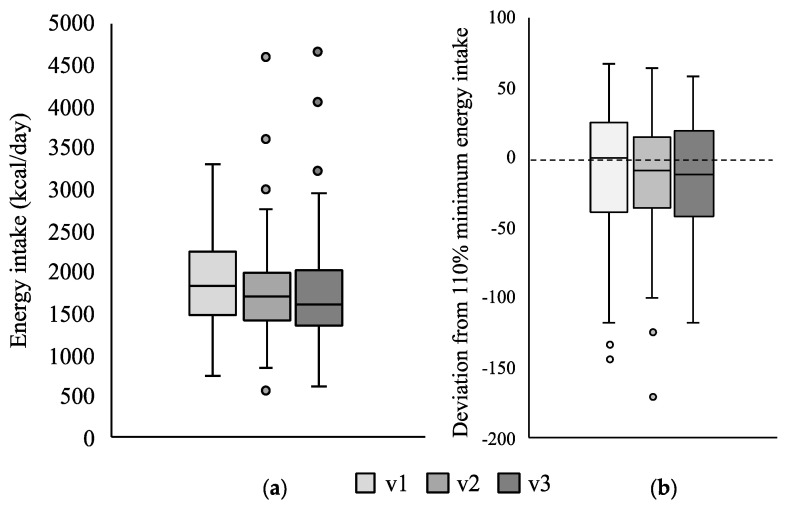
Change in daily energy intake along the three study visits (v1, baseline; v2, month 3; v3, month 6) in the study cohort (*n* = 84). (**a**) expressed as median kcal/day; (**b**) expressed as deviation% from minimum recommended daily energy intake (110% with respect to age and gender-matched populations, represented by horizontal dotted line) [3].

The median reported daily energy intake at baseline and after three and six months was, respectively, 1803, 1690 and 1657 kcal (Figure 1a). Energy intake was also expressed as deviation from the minimum 110% energy recommendation [3] (Figure 1b): at v1, participants had a median intake of −0.2% (−37.2, 24.1) with respect to the minimum recommendation, and this gap increased although not significantly at visits v2 and v3, median values being −9.5% (−36.2, 14.2) and −11.9% (−41.4, 19.0), respectively.

When assessing compliance to the minimum recommendation for the different age groups, the same pattern was observed. However, the gap in daily energy intake along the study period was mainly seen in girls rather than in boys, although the changes were again not statistically significant (girls: from −9% (−47, 17) to −22% (−57, 14); boys: from 5% (−32, 26) to −2% (−24, 31)). Looking at the centres individually, the same trends were seen. Change in energy intake was not significantly associated with z-scores for weight, height and BMI, even when considering age and gender.

### 3.2. Change in Macronutrient Distribution

**Figure 2:**
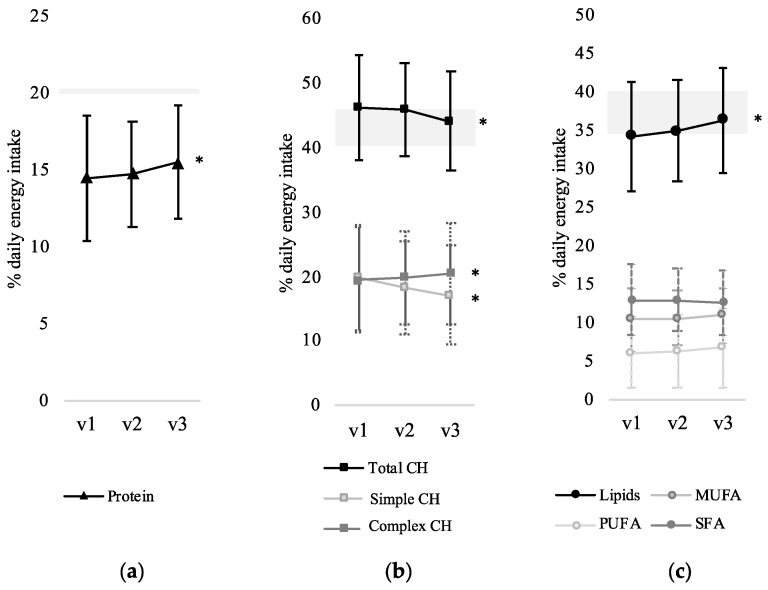
Changes in macronutrient intake during the 6-months period of use of the self-management mobile app in the study cohort (*n* = 84): (**a**) protein, (**b**) carbohydrate and (**c**) lipids. Grey areas represent the range of the recommended intake according to Turk et al. (2016) [3]. According to the WHO guidelines, simple CH (sugar) intake should not be higher than 5% energy intake, and SFA should not exceed 10%. * statistically significant change (*p* < 0.05) between visit in month 0 (v1) and month 6 (v3). Original to this manuscript).

The contribution of the three macronutrients to the total daily energy intake more closely approached the guidelines’ recommendations after 6 months of app use by decreasing total carbohydrate intake along with increasing protein and lipids (Figure 2). The net increase or decrease in nutrient intake and its statistical effects are presented in Table 1.

At study baseline (v1), macronutrient distribution was characterized by a mean (SD) of 46.2% (8.2) total carbohydrates, 14.5% (5.1) protein and 34.3% (7.1) lipids, while the results after 6 months (v3) significantly changed to 44.1% (7.7), 15.5% (3.7) and 36.4% (6.9), respectively. Focusing on the type of carbohydrates, complex carbohydrates’ intake gradually increased from 19.4% (7.8) to 20.5% (7.4) at the expense of a decrease in simple carbohydrates (sugar) consumption from 19.7% (8.6) to 17.1% (8.8). Although not statistically significant, slight changes in the type of lipids were observed, with a decreasing intake of SFA and an increasing intake of PUFA and MUFA. The rest of the assessed items, e.g., fibre, iron, calcium and sodium, did not show relevant changes along the study period.

**Figure 3:**
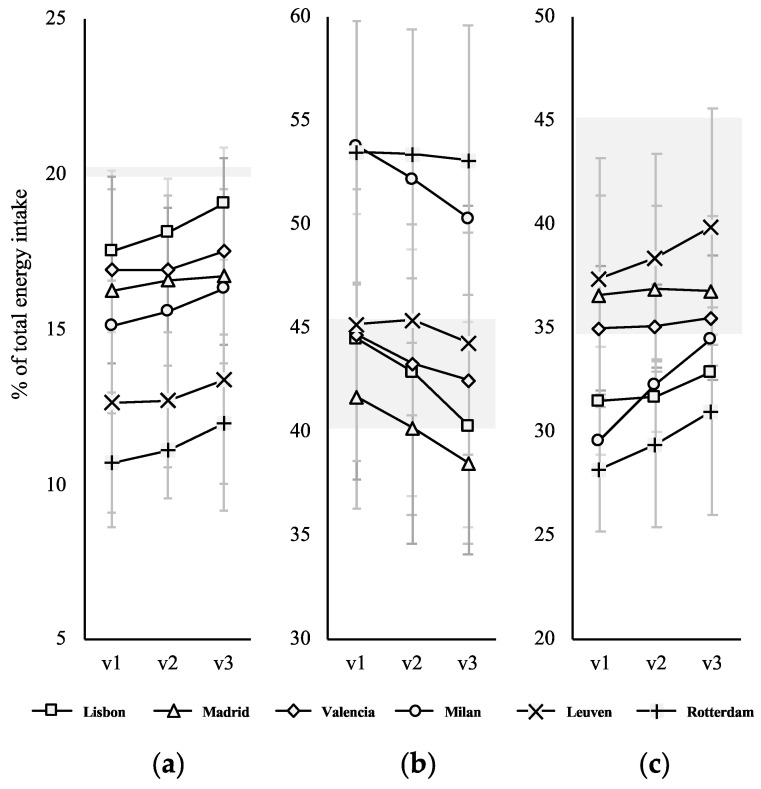
Evolution of macronutrient intake during the 6-months period of use of the self-management mobile app in the six participating European centres: (**a**) protein, (**b**) carbohydrates and (**c**) lipid. Grey areas represent the range of the recommended intake according to Turck et al. (2016) [3].

The changes experienced in the individual centres were similar to those in the whole cohort. Protein intake increased in all centres (Figure 3a). However, at no point did the mean intake reach the recommended 20% of total energy intake. Lisbon was the centre approaching this the most, with a mean of 19% (1.8) at v3. Lisbon, along with the other southern centres (Madrid and Valencia), were those registering the highest protein intake from the baseline, but in Madrid and Valencia, the increase was lower than 1%. In Milan, protein intake changed from mean 15.1% (1.6) to 16.2% (2.0). The Northern centres, Leuven and Rotterdam, were those starting with the lowest protein intakes, 12.6% (3.9) and 10.7% (1.6), respectively, which increased more in Rotterdam (to 12.0%) than in Leuven (to 13.3%).

Regarding total carbohydrates, Milan and Rotterdam were the centres registering the highest intakes (Figure 3b), which decreased in both of them, but not enough to reach the recommended range. The other centres had a lower initial intake of total carbohydrates, below 45%, with the lowest value recorded in Madrid (41.7% (5.4)). All of them showed a decrease of at least −2% at month 6 (v3).

Focusing on lipids (Figure 3c), three centres were within the recommendation of 35–40% of the total energy intake at v1: Leuven, Madrid and Valencia. In all of them an increase of 1–2% was registered at month 6 in v3. In the other three centres, a similar increase was observed, although none reached a mean intake within the recommended range.

### 3.3. Change in Dietary Intake

**Figure 4:**
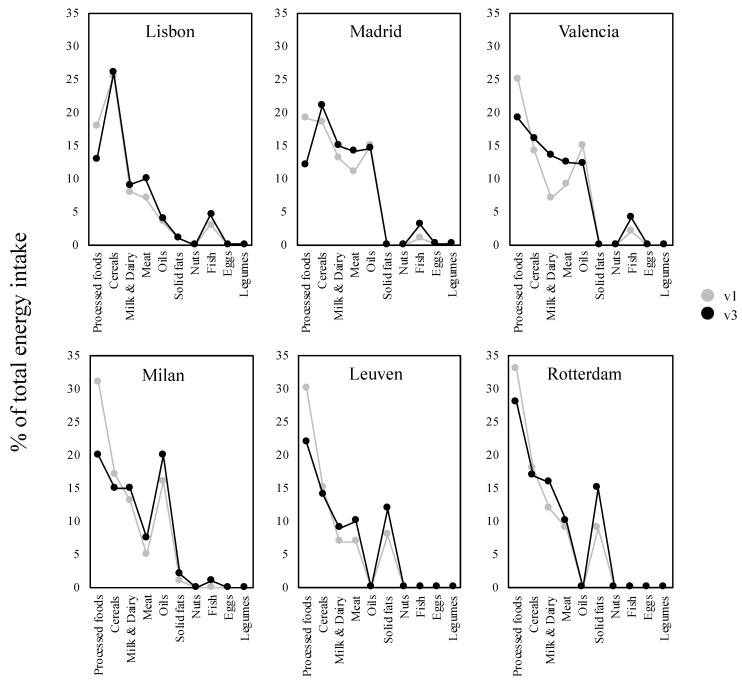
Relative contribution of food groups to total daily energy intake at study baseline (v1) and after the intervention (v3) of the clinical trial in the six European participating centres. Original to this manuscript.

In order to explain energy and nutrient intake changes in the study cohort, the contribution of food groups to total daily energy intake was assessed. As shown in Figure 4, ultra-processed foods showed the largest drop in contribution to total energy intake in all centres (from −5% in Lisbon and Rotterdam to −11% in Milan), but remained the main source of energy after the 6-month intervention, except in Lisbon, where already from baseline the cereals group contributed the most, and in Madrid, where cereals surpassed ultra-processed foods and became the main contributor to energy intake. In contrast, the increase of energy coming from meat (1–3.5%) and dairy products (1–6.5%) was another common pattern observed in all the centres. On the other hand, food groups that did not change their contribution to total daily energy intake were eggs and legumes, which remained practically negligible. A moderate increase in fish intake was only noticed in Lisbon, Madrid, Valencia and Milan (1–2.5%). Regarding lipid sources, oils (especially olive oil) made a relevant contribution to the total energy intake in the same centres, especially in Milan (where it reached 20% of total energy intake at v3). Solid fats (especially butter and margarine) were the lipid sources contributing the most to total energy intake and underwent a higher increase compared to oil in the other centres. Finally, fruit and vegetables are not represented in this analysis, as their low calorie content is negligible to contribute to total daily energy intake. Considering these groups as number of times consumed a day at baseline and after 6 months, similar numbers were obtained, in the ranges of 1.2–1.5 times a day for fruit and 1.1–2.6 times a day for vegetables.

## 4. Discussion

In the present study, a 6-month thorough monitoring of dietary intake in a paediatric patient population with CF was accomplished. Food recording was supported by a self-management mobile app that included resources to empower patients to meet the current CF-specific nutrition guidelines. At the end of the intervention, energy intake slightly decreased, and macronutrient distribution better approximated recommendations, while nutritional status indicators did not change. However, the detected changes were still insufficient to meet the standards of a standard healthy and adequate diet.

A reinforced message in the current ECFS/ESPGHAN/ESPEN nutrition guidelines for CF is that energy intake should be achieved by supplying healthy sources of fat and carbohydrates [3]. This recommendation is aligned with the strategies posed by the global health authorities. Of note, the WHO strongly recommends a sugar intake of <5% and SFA <10% of the daily energy intake for the general population [10]. The difficulty in limiting sugar and SFA intakes relies on their presence, and in large amounts, in many of the food products that can be found in the supermarkets, i.e., ultra-processed foods [17].

These foods have displaced the consumption of fresh foods and have alienated consumers from healthy dietary patterns, such as the Mediterranean diet [18]. The reasons for the preference for ultra-processed foods rely on its low cost, readiness to consume and palatability [19]. Moreover, they were usually supported even by CF teams because of their high energy and fat content. Thus, a strong emphasis is needed on the nutritional education of patients with CF. This should include counselling by a dietitian and providing adequate resources for appropriate self-directed dietary choices. Our results depict a positive trend towards reduced sugar intake, along with a slight decrease in the energy intake, that can be attributed to significantly reduced ultra-processed products intake. Despite the concomitant increase in dairy and meat products, in which the type of fat is mainly saturated, a decrease in SFA intake was noted. The higher presence of meat and dairy products, in turn, probably explains the significant increase in protein and lipid intake.

The unhealthy dietary pattern observed in the present study, which is characterized by low consumption of recommended food groups like nuts, fish or legumes, is not limited to the CF populations but is also present in the general population [20,21,22]. Despite few in-depth studies assessing the quality of the diet in CF patients being available, the results of the present study are in agreement with those reported by Woestenenk et al. in 2014 and Sutherland et al. in 2017 [7,8]. If one considers the nutritional intake of a similar cohort of CF patients recruited by the same participating centres three years before starting the clinical trial, a slight improvement in nutrient and dietary intake was observed [9]. In the referred cross-sectional observational study, the median simple carbohydrate intake was comparable (19.6%, range 14.0–24.9%), and saturated fat represented 13.1% of the total energy intake (11.1–15.4).

According to a previous study on user-experience conducted in patients participating in the trial, the follow-up of nutrient intake was perceived as one of the most useful features (after the prediction of the dose of enzyme supplements) [23]. Results of that study may help to understand the findings reported in this paper. Although most of the participants reported increased knowledge about nutrition and awareness about food intake, some of them perceived nutritional goals as difficult to achieve and decided to avoid looking at them. However, some parents reported that this feature promoted dietary changes, suggesting that patients’ awareness and follow-up of daily goals could have motivated dietary changes towards increased protein and lipid intake along with decreased carbohydrates, but this still did not result in reducing ultra-processed foods to the desired extent.

Our study has many limitations. The collection of study data relied on patients’ self-reporting. Food recording could have gone down over time because of app fatigue, causing skipping of recording of some meals. It is worth noting that the main motivation for patients to record meals was obtaining the optimal dose of enzymes. Meals that were consumed regularly could have informed patients about the enzyme dose needed already, with loss of motivation for repeated food recording. This fact could explain at least in part the decrease in total reported energy intake towards the end of the study. Nonetheless, to reduce this limitation, only patients with at least 5 meals registered a day, for three days in a week and at least once per study visit were considered. Another limitation of the present study is that the study design was set up to evaluate change in quality of life as a primary outcome, and not specifically to evaluate change in nutrient or food groups intake. On the other hand, the strengths of the study include the large amount of data, the three time points of data collection, the robust and validated system to process food records and the monitoring of dietary and nutrient intake by healthcare professionals.

There are only few interventional studies in the literature regarding nutritional education to promote dietary changes [24,25]. Most of the interventions to improve nutritional status in patients with CF relate to non-dietary actions, particularly tube feeding, which has repeatedly confirmed its effectiveness [26]. Tube feeding is recommended in specific situations, when stunting or wasting cannot be recovered only with diet, so that other non-invasive actions could be encouraged for the routinely and daily patient self-managed treatment.

In the clinical practice, in order for patients to adhere to a healthy diet while also meeting the recommended intake according to guidelines, the use of a mobile app that supports self-assessment of food and nutrient intake and provides nutritional education and practical recommendations could be helpful. In parallel, the use of this tool could help healthcare professionals to retrieve dietary intake of patients for accurate and thorough dietary assessment, possibly replacing regular paper food records. Through this system, consultation time would be more efficient, saving time in calculating food records. However, as evidenced in this study, the assessed system would still need refinement in order to prevent from decline in energy intake and reinforce the reduction of ultra-processed foods intake. Possible strategies to improve food recording through app use in the long run would include simpler food recording (e.g., voice commands or food scanning) or a simplified and more user-friendly identification of food items.

## 5. Conclusions

The present study can be considered as a first attempt towards establishing a novel system to support and evaluate nutritional therapy in CF by means of mHealth (or mobile health) technology. Its incorporation in the frame of a clinical trial implied challenges due to technological literacy requirements, both for healthcare professionals and patients’ caregivers. Moreover, this first prototype of the app was not fully satisfying patients’ needs, despite the fact that it was co-developed with them [13]. In particular, the food recording feature was identified as needing improvement [23]. However, the results observed after 6 months of use suggest that, despite initial drawbacks, the refinement of the system could improve adherence and nutritional outcomes. Further technological development and a longer period of follow-up in the context of a long-term observational study with this self-management app could improve the results presented in this study. In addition, to validate the benefits of using the app for improving dietary intake, a controlled trial with ideally a longer period of follow-up should be carried out.

## Figures and Tables

**Table 1:** Change in macronutrient contribution (% of total daily energy intake) after the 6-month intervention with the self-management mobile app.

% of Total Energy Intake	Difference from v1 to v3	Odds Ratio, *p*-Value	95% Confidence Interval
% Protein intake	+1.0%	1.09, *p* = 0.001	(1.03, 1.15)
% Lipid intake	+2.0%	1.09, *p* = 0.02	(1.01, 1.18)
% SFA intake	−0.4%	0.97, *p* = 0.42	(0.90, 1.04)
% MUFA intake	+0.6%	1.05, *p* = 0.28	(0.96, 1.14)
% PUFA intake	+0.6%	1.09, *p* = 0.22	(0.95, 1.25)
% CH intake	−2.5%	0.88, *p* = 0.001	(0.81, 0.95)
% Simple CH intake	−2.4%	0.81, *p* < 0.001	(0.73, 0.89)
% Complex CH intake	+1.0%	1.03, *p* = 0.003	(1.01, 1.07)

SFA: saturated fatty acids; MUFA; monounsaturated fatty acids; PUFA; polyunsaturated fatty acids; CH: carbohydrates.

## Supplementary Materials

The following are available online at https://www.mdpi.com/article/10.3390/nu13061801/s1, Figure S1: Home menu of the self-management app, Figure S2: Food recording through the “food diary” feature, Figure S3: Self-monitoring of nutrient intake through the “Goals” feature, Figure S4: Overview of the professional web tool.
