# Supplementary material for: Change in Nutrient and Dietary Intake in European Children with Cystic Fibrosis after a 6-Month Intervention with a Self-Management mHealth Tool
Source: Nutrients. 2021 May 26;13(6):1801. doi: 10.3390/nu13061801 (PMC8229611; doi:10.3390/nu13061801)

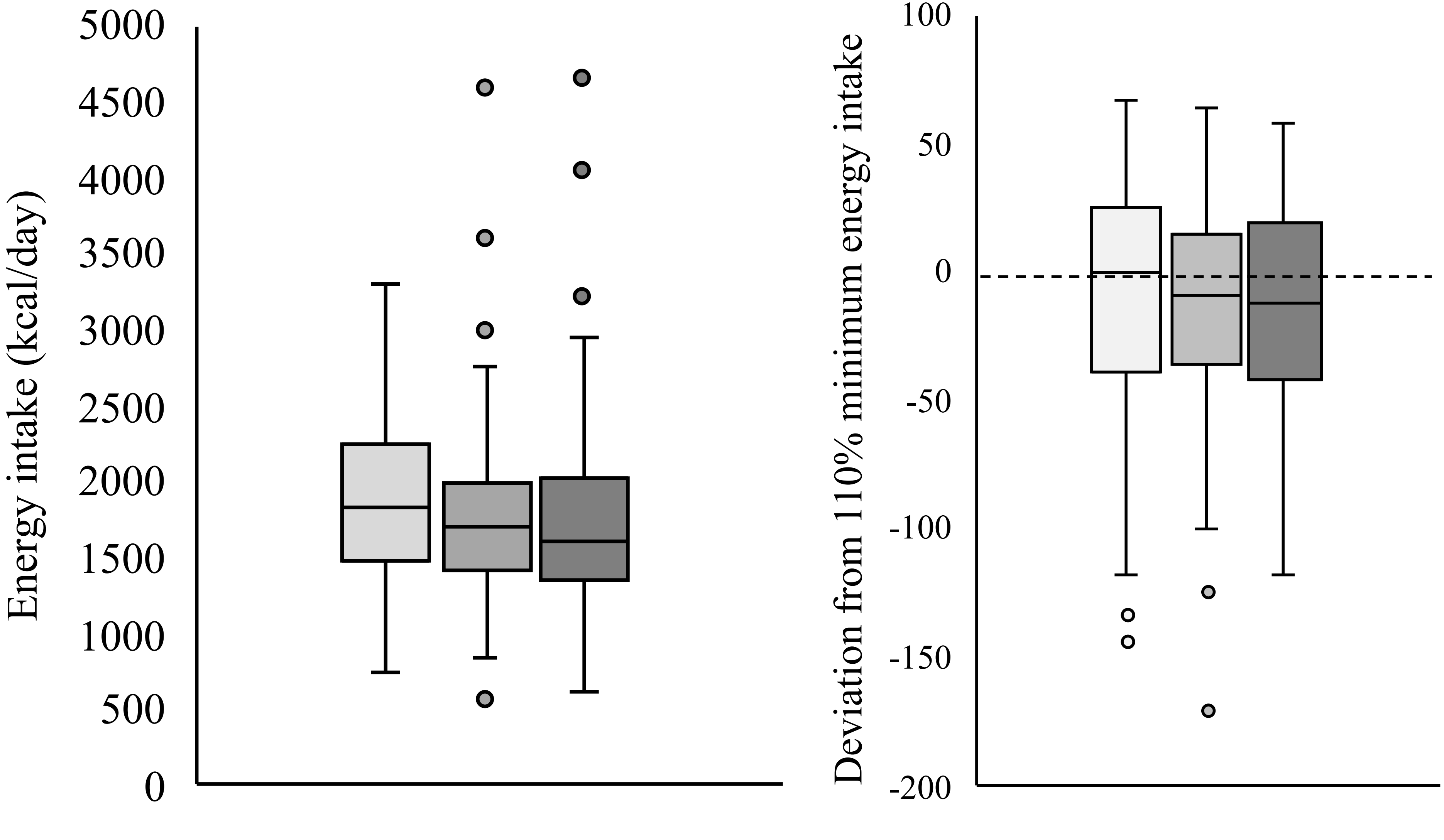
Energy intake (kcal/day)
5000
4500
4000
3500
3000
2500
2000
1500
1000
500
0
Deviation from 110% minimum energy intake
100
50
0
-50
-100
-150
-200
v1
v2
v3

(**a**) (**b**)

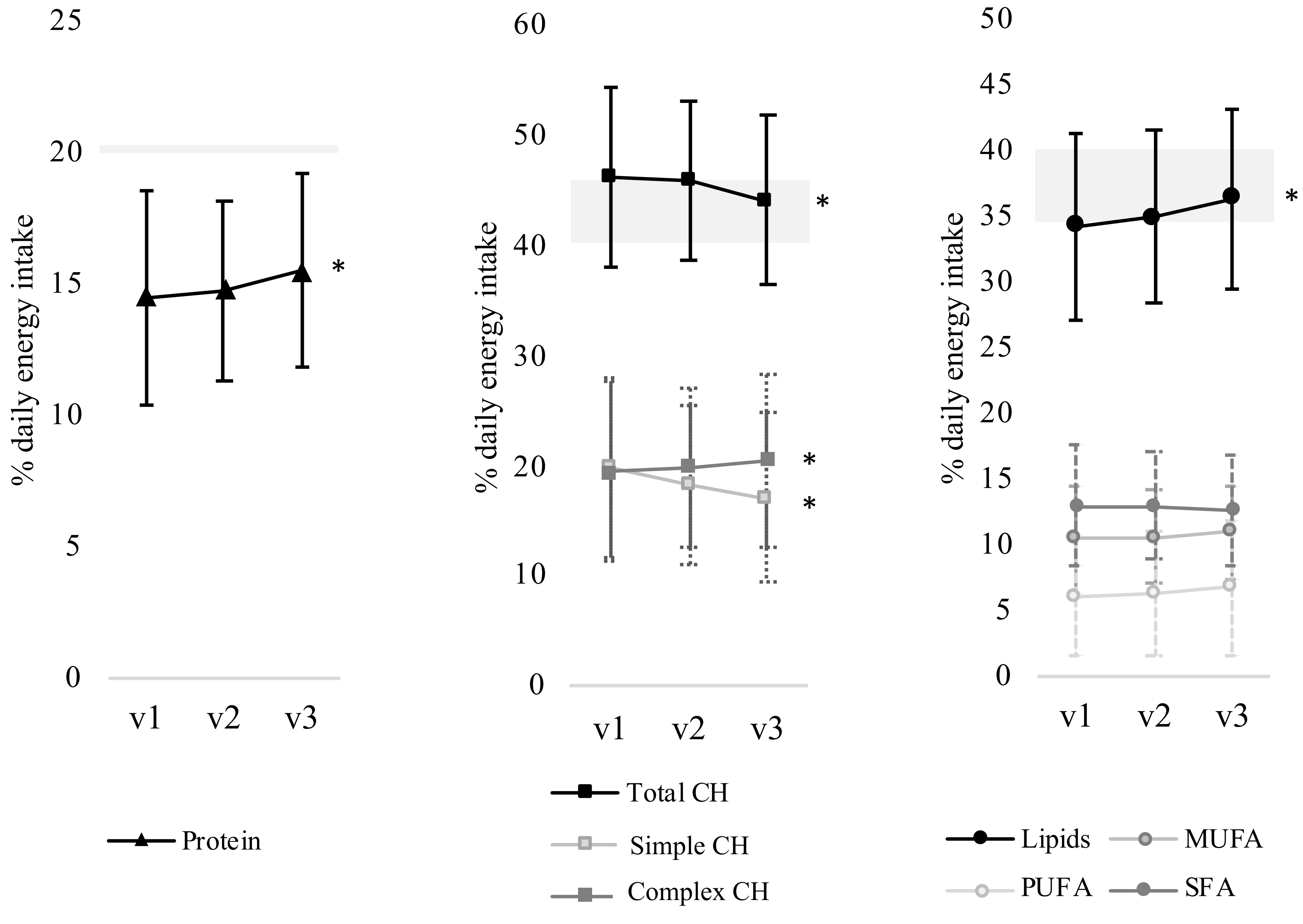
% daily energy intake
25
20
15
10
5
0
v1
v2
v3
*
Protein
% daily energy intake
60
50
40
30
20
10
0
v1
v2
v3
*
*
*
Total CH
Simple CH
Complex CH
% daily energy intake
50
45
40
35
30
25
20
15
10
5
0
v1
v2
v3
*
Lipids
MUFA
PUFA
SFA

(a) (b) (c)

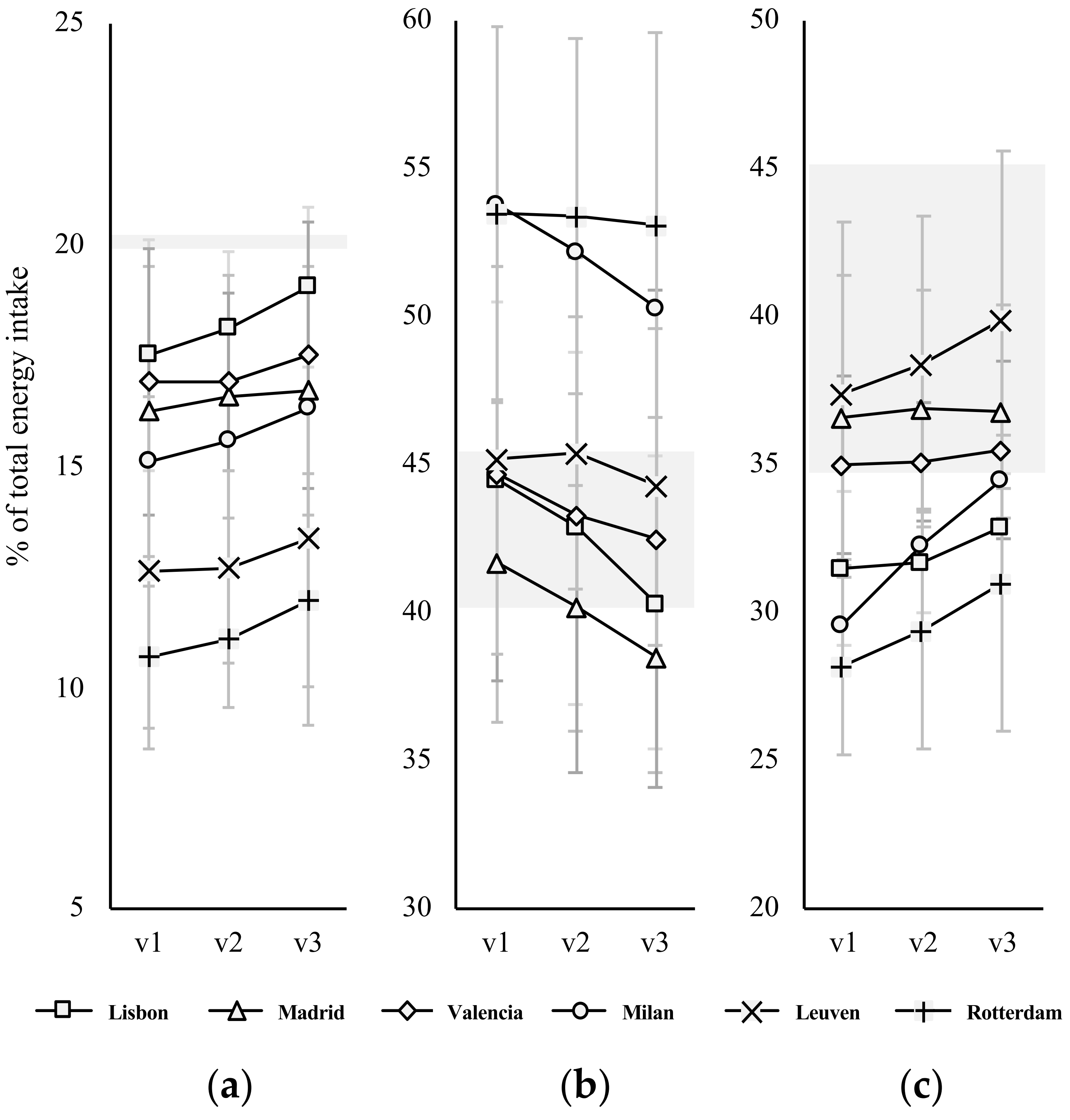
% of total energy intake
25
20
15
10
5
60
55
50
45
40
35
30
50
45
40
35
30
25
20
v1
v2
v3
Lisbon
Madrid
Valencia
Milan
Leuven
Rotterdam
(a)
(b)
(c)

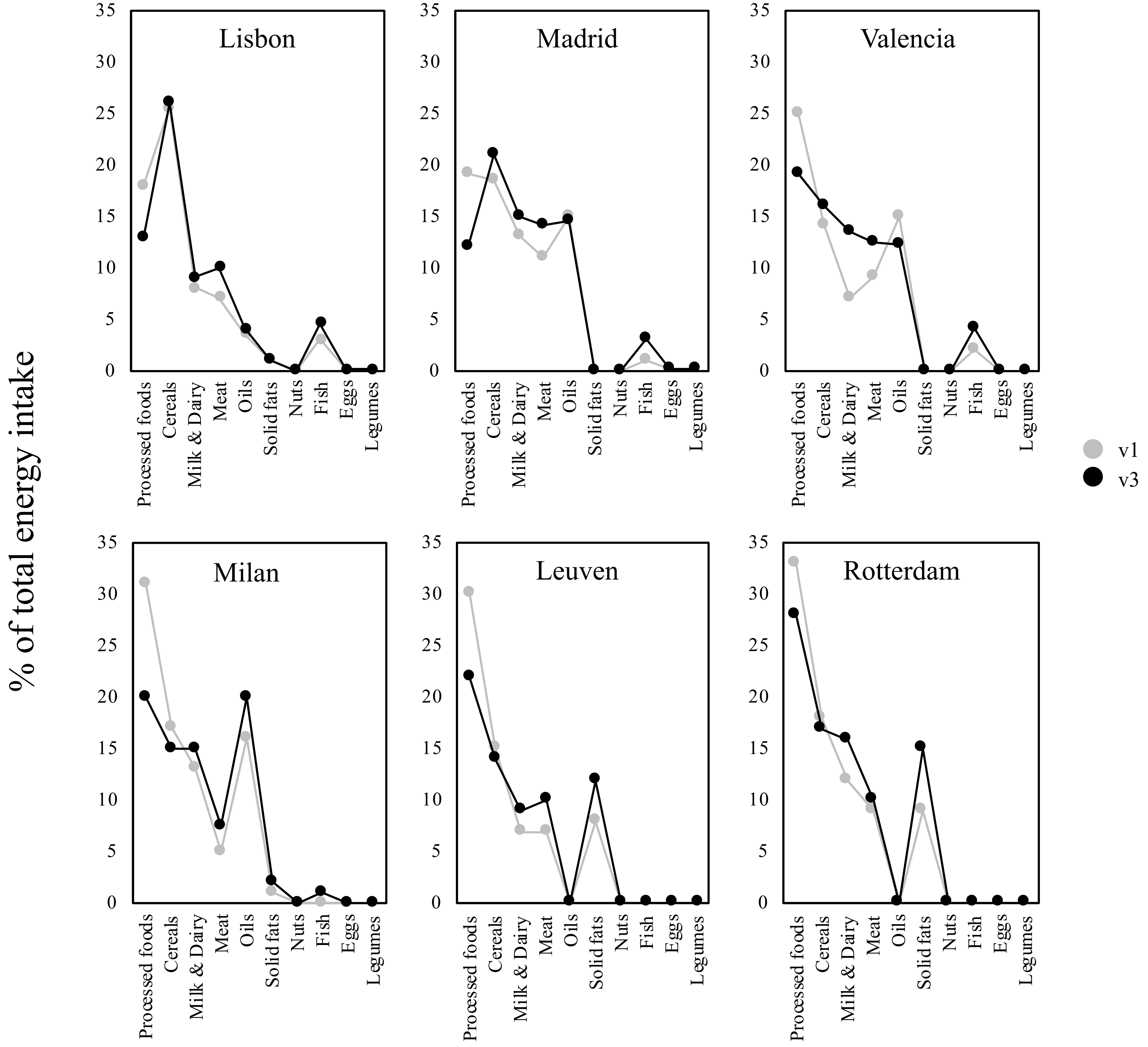
% of total energy intake
Lisbon
Madrid
Valencia
Milan
Leuven
Rotterdam
Processed foods
Cereals
Milk & Dairy
Meat
Oils
Solid fats
Nuts
Fish
Eggs
Legumes
0
5
10
15
20
25
30
35
v1
v3

Figure 4

Figure S1

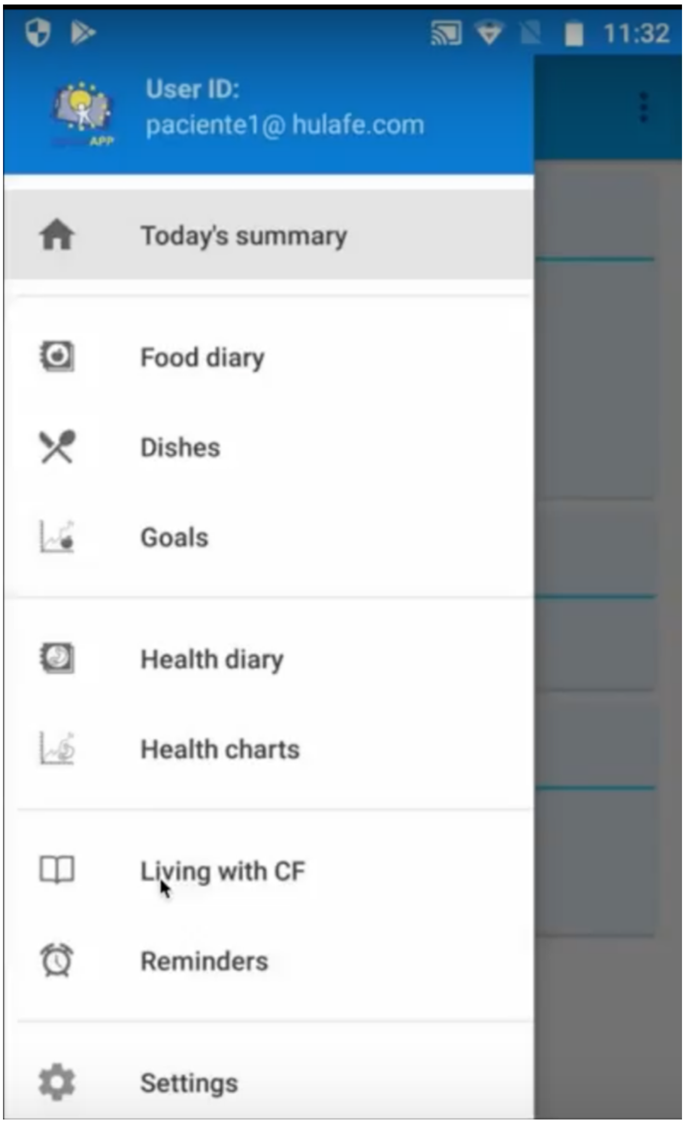
11:32
User ID:
paciente1@ hulafe.com
Today's summary
Food diary
Dishes
Goals
Health diary
Health charts
Living with CF
Reminders
Settings

Figure S2

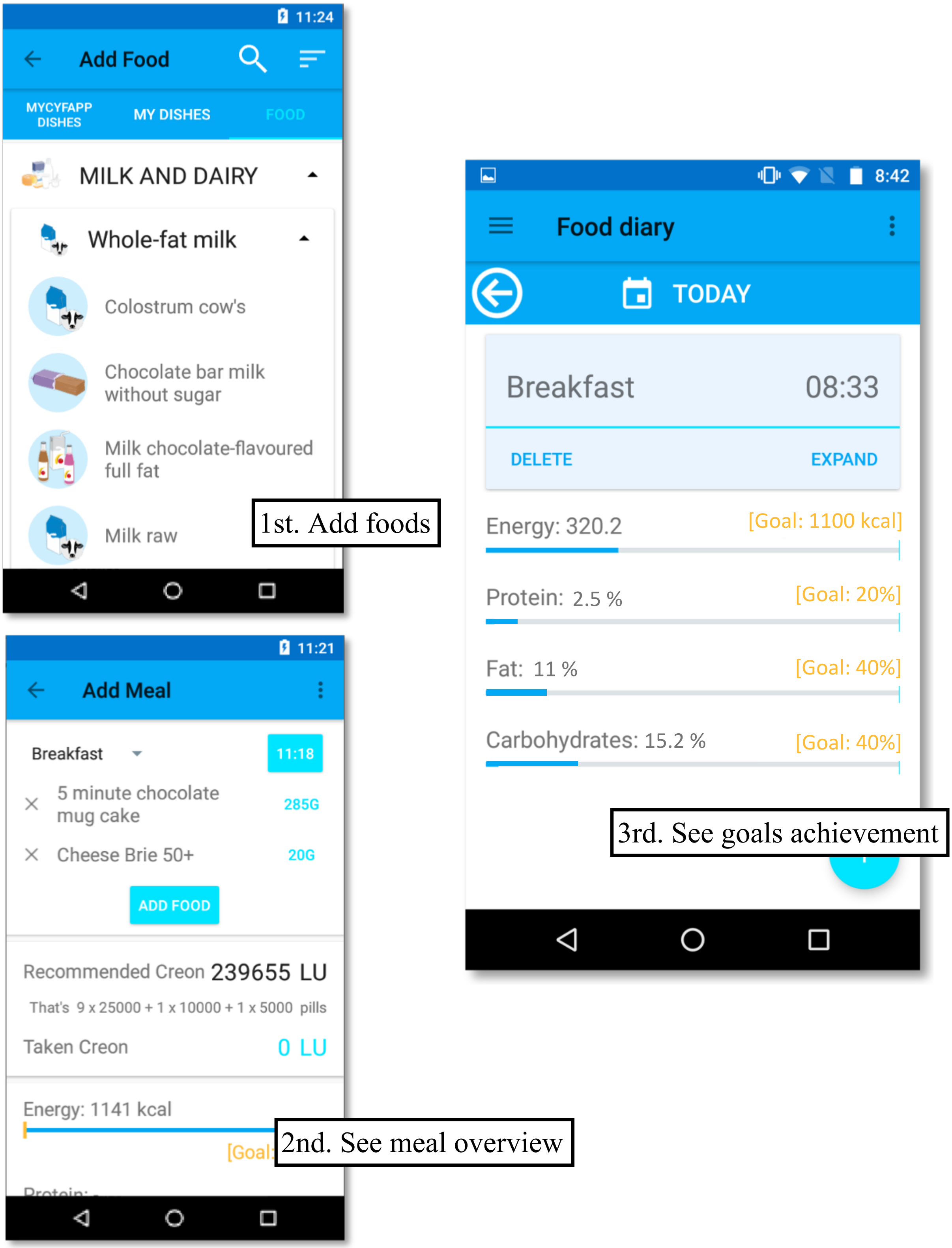
11:24
Add Food
MYCYFAPP DISHES
MY DISHES
FOOD
MILK AND DAIRY
Whole-fat milk
Colostrum cow's
Chocolate bar milk without sugar
Milk chocolate-flavoured full fat
Milk raw
1st. Add foods
11:21
Add Meal
Breakfast
11:18
5 minute chocolate mug cake
285G
Cheese Brie 50+
20G
ADD FOOD
Recommended Creon 239655 LU
That's 9 x 25000 + 1 x 10000 + 1 x 5000 pills
Taken Creon
0 LU
Energy: 1141 kcal
2nd. See meal overview
8:42
Food diary
TODAY
Breakfast
08:33
DELETE
EXPAND
Energy: 320.2
[Goal: 1100 kcal]
Protein: 2.5 %
[Goal: 20%]
Fat: 11 %
[Goal: 40%]
Carbohydrates: 15.2 %
[Goal: 40%]
3rd. See goals achievement

Figure S3

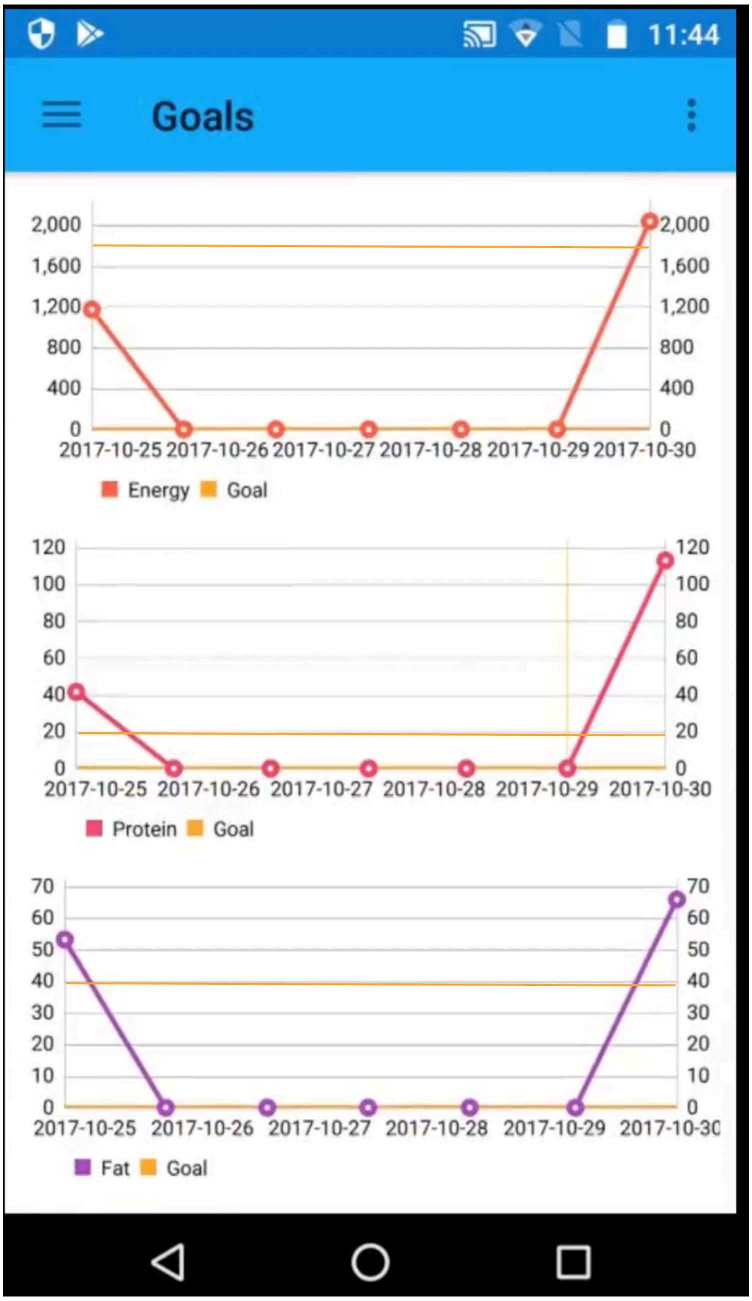
11:44
Goals
2,000
1,600
1,200
800
400
0
2017-10-25 2017-10-26 2017-10-27 2017-10-28 2017-10-29 2017-10-30
Energy Goal
120
100
80
60
40
20
0
2017-10-25 2017-10-26 2017-10-27 2017-10-28 2017-10-29 2017-10-30
Protein Goal
70
60
50
40
30
20
10
0
2017-10-25 2017-10-26 2017-10-27 2017-10-28 2017-10-29
Fat Goal

Figure S4

Patient profile (upper part) and recommendation messages (lower part)

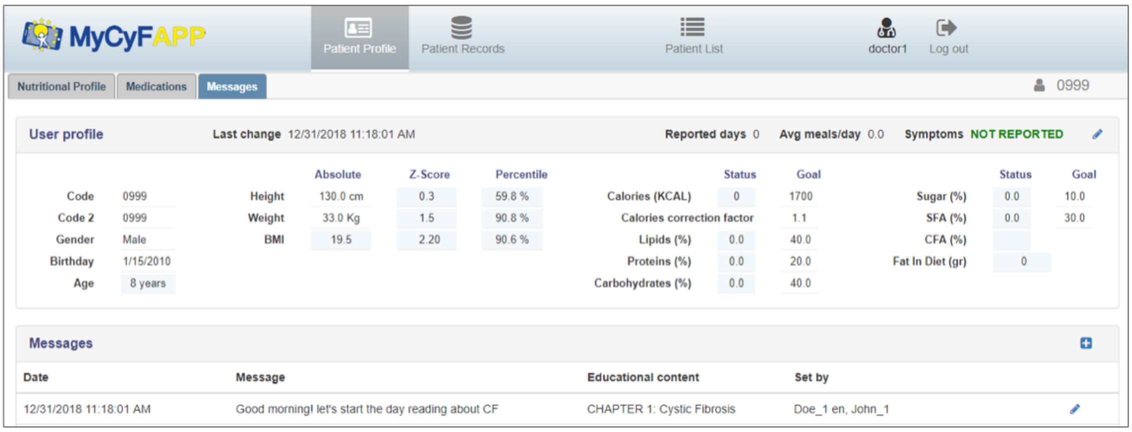

Patient records: nutritional intake

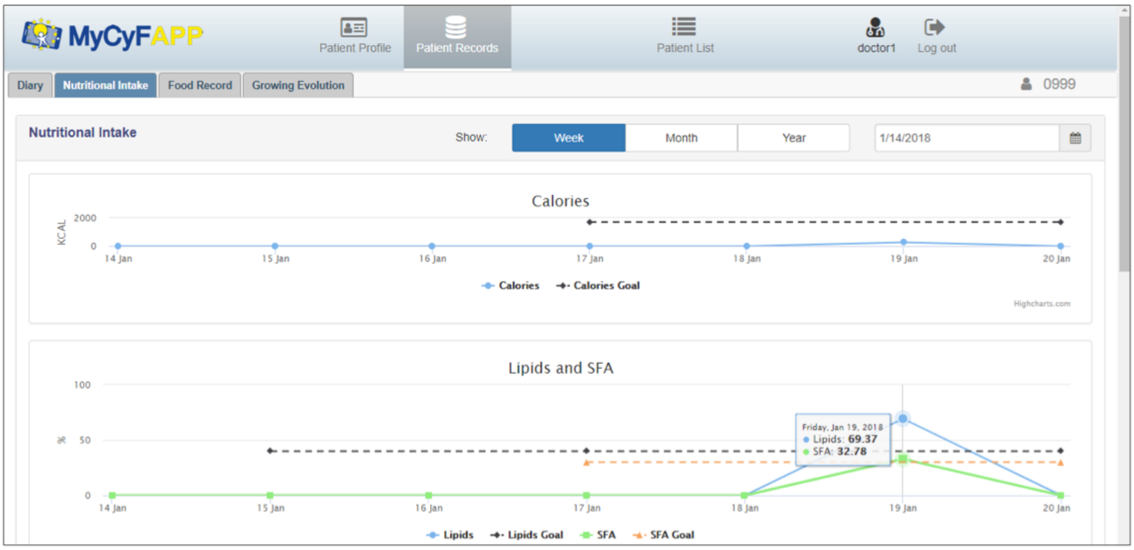

Patient records: food records over time

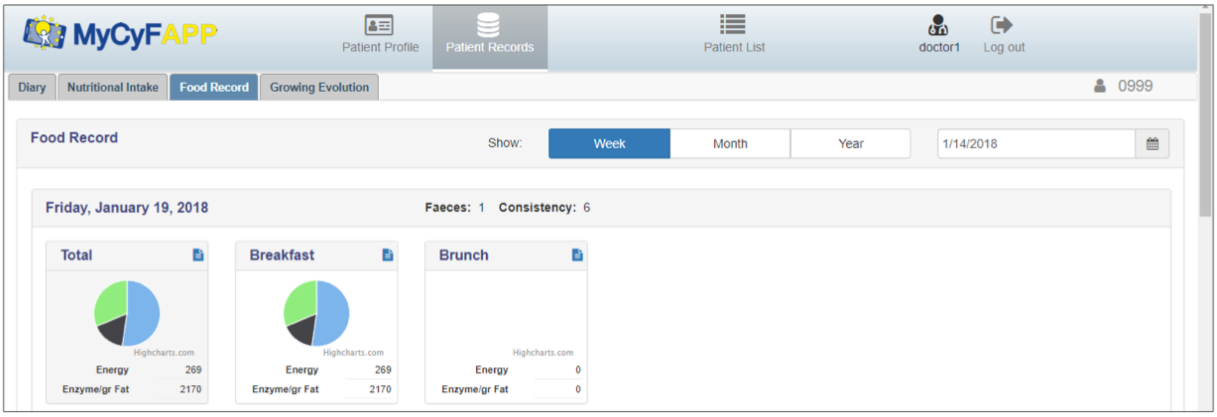

Supplement: Supplementary file 1 [file nutrients-13-01801-s001.zip › nutrients-1184539-suppl.pdf]
